# Post-Graduate Ophthalmology at Oxford

**Published:** 1909-04-10

**Authors:** 


### POST-GRADUATE OPHTHALMOLOGY AT OXFORD

The sixth annual post-graduate course on ophthalmology in connection with the Faculty of Medicine of the University of Oxford has been arranged from July 5 to 17. The course is designed to be a practical adjunct to the reading of ophthalmological text-books. The first part will be concerned chiefly with demonstrating the practical examination of eye patients, the use of the ophthalmoscope, and refraction. The second part of the course will be more specialised, and lectures will be delivered by various ophthalmic surgeons. Upwards of 500 cases will be present for demonstration, and these will include many especially suitable for more advanced and experienced students and practitioners of ophthalmology. The fee for the course is £5 5s. Medical men attending will be provided with board and residence at Keble College at 7s. 6d. a day during the second week, but during the first week lodgings must be secured. Further particulars can be obtained from Mr. Robert W. Doyne, of the Ophthalmological Department of the University of Oxford, 30 Cavendish Square, London, W.

